# Blockade of the TLR4–MD2 complex lowers blood pressure and improves vascular function in a murine model of type 1 diabetes

**DOI:** 10.1038/s41598-020-68919-x

**Published:** 2020-07-21

**Authors:** Amanda Almeida de Oliveira, Josemar Faustino, R. Clinton Webb, Kenia Pedrosa Nunes

**Affiliations:** 1 Department of Biomedical and Chemical Engineering and Sciences, Florida Institute of Technology, Melbourne, USA; 2 Department of Computer Engineering and Sciences, Florida Institute of Technology, Melbourne, USA; 3 Department of Cell Biology and Anatomy, University of South Carolina, Columbia, USA

**Keywords:** Type 1 diabetes, Vascular diseases

## Abstract

While the pathogenesis of diabetes-induced high blood pressure (BP) is not entirely clear, current evidence suggests that Toll-like receptor 4 (TLR4) is a key player in the mechanisms associated with hypertension. However, it is unknown whether this receptor affects BP under type 1 diabetes. Likewise, there is insufficient knowledge about the role of TLR4 in diabetes-associated vascular dysfunction of large arteries. To narrow these gaps, in this study, we investigated if blockade of the TLR4-MD2 complex impacts BP and vascular function in diabetic rats. We injected streptozotocin in male Sprague Dawley rats and treated them with a neutralizing anti-TLR4 antibody for 14 days. BP was directly measured in conscious animals at the end of the treatment. In another set of experiments, we excised the aorta from control and diabetic animals, and measured TLR4 and MD2—a co-receptor that confers functionality to TLR4—levels by Western blotting. We also performed functional studies and evaluated ROS levels with and without a pharmacological inhibitor for TLR4 as well as for MD2. Additionally, we scrutinized a large human RNA-Seq dataset of aortic tissue to assess the co-expression of TLR4, MD2, and subunits of the vascular NADPH oxidases under diabetes and hypertension. We report that (a) chronic blockade of the TLR4–MD2 complex lowers BP in diabetic animals; that (b) type 1 diabetes modulates the levels of MD2 expression in the aorta, but not TLR4, at least in the conditions evaluated in this study; and, that (c) acute inhibition of TLR4 or MD2 diminishes vascular contractility and reduces oxidative stress in the aorta of these animals. In summary, we show evidence that the TLR4–MD2 complex is involved in the mechanisms linking type 1 diabetes and hypertension.

## Introduction

A significant concern in public health, diabetes affects 1 in every 11 adults worldwide^1^. The disease is often associated with other chronic disorders such as hypertension^2^. The American Diabetes Association (ADA) estimates that between 20 and 60% of diabetic patients become hypertensive^3^. Type 1 diabetes-induced hypertension occurs at a relatively young age and affects both sexes at the same rate^4^. Prolonged exposure to the condition increases the likelihood of developing cardiovascular diseases (CVDs), which is the single-most cause of death in diabetic patients^5^. While the etiology of high blood pressure (BP) in these patients is not entirely clear, the current literature suggests an influence of the immune system in the pathophysiology of hypertension^6,7^. Similarly, recent studies indicate an active involvement of the immune system in the development of vascular diabetic complications^8^.

There is evidence that, over time, high glucose impairs the balance between vasoconstrictors and vasodilators^9^, which alters the paracrine control of the muscle layer leading to hypercontractility in small and large vessels. Interestingly, previous studies have demonstrated that Toll-like receptor 4 (TLR4)—a receptor that is part of the innate immune system—mediates vascular dysfunction in small resistance arteries in disease models of diabetes^10^ and hypertension^11^. Additionally, TLR4 differentially affects BP in hypertensive animals^12^, although its blockade reduces the production of reactive oxygen species (ROS) and inflammatory markers in major organs that control BP^13^. However, there is insufficient information about the role of TLR4 in type 1 diabetes-induced vascular dysfunction of large arteries, and it is unknown whether TLR4 impacts BP under type 1 diabetes. Therefore, further investigation of this pathway could elucidate TLR4 as a common target between the two diseases.

TLR4 physically interacts with the co-adaptor myeloid differentiator factor 2 (MD2)^14^, forming a functional heterodimer in the innate immunity machinery. Recent studies have also highlighted a role for MD2 in chronic conditions as the pharmacological blockade of MD2 impairs the renin-angiotensin system (RAS) in the diabetic kidney^15^. The RAS plays a decisive function in BP regulation, and its main active peptide, angiotensin II (AngII), activates TLR4 by binding the co-adaptor MD2^16,17^. Additionally, in diet-induced obesity, the deletion of MD2 protects against remodeling and oxidative stress in the vasculature^18^. Yet, the role of MD2 in type 1 diabetes-associated vascular dysfunction of large arteries is unknown.

Therefore, in this work, we examined whether chronic blockade of the TLR4–MD2 complex with a neutralizing antibody affects BP under type 1 diabetes. We also performed functional studies in isolated thoracic aorta in the presence and absence of an inhibitor for TLR4 and its co-adaptor MD2 independently. ROS were indirectly evaluated with a fluorescent probe in cultured vascular smooth muscle cells (VSMCs) subjected to high glucose as well as in the aorta of rats with diabetes induced via streptozotocin (STZ) injection. Additionally, we scrutinized a large human RNA-Seq dataset of aortic tissue to assess the co-expression of TLR4 and MD2 as well as subunits of the vascular NADPH oxidases under diabetes and hypertension. We report that (a) systemic blockade of the TLR4-MD2 complex lowers mean arterial pressure (MAP) in diabetic animals; that (b) type 1 diabetes modulates the levels of MD2 in the aorta, but not of TLR4, at least in the conditions evaluated in this study; and, that (c) acute inhibition of this receptor or its co-adaptor improves aortic vascular function by reducing vascular contractility and oxidative stress. Altogether, these findings highlight the TLR4–MD2 complex as a novel therapeutic target in type 1 diabetes-induced hypertension.

## Material and methods

### Animals

All animal procedures performed in this study are in accordance with the Guide for the Care and Use of Laboratory Animals from the National Institutes of Health and were reviewed and approved by the Institutional Animal Care and Use Committees of Augusta University and Florida Institute of Technology. Sprague Dawley male rats (200–220 g) from Charles River Laboratory and Taconic Biosciences were used in our chronic and acute treatments, respectively. Animals were housed under alternating light/dark photoperiods of twelve hours each, and were offered both standard chow diet and water ad libitum. Upon a 48 hour acclimation period, the treatment group of rats were injected with a single dose of STZ (65 mg/kg) leading to diabetes^19^. Diabetic animals, glucose levels equal to or greater than 250 mg/dl, were kept in the animal facility for 28 days.

#### Chronic treatment with a neutralizing antibody

During the last 14 days of the treatment, we injected half of the diabetic rats in the treatment group with an anti-TLR4 antibody, raised against the TLR4–MD2 complex (Santa Cruz Biotechnology, sc13591; 1 μg/daily). We diluted the antibody in a sterile saline solution and determined the dosage, route of administration (intraperitoneal), and frequency based on previous work^11^. Control animals (CTL) were injected only with vehicle as a non-specific IgG antibody does not affect this physiological parameter^11,20^. We randomly selected groups of animals in the following fashion: CTL, STZ, CTL treated with an anti-TLR4 antibody, and STZ treated with an anti-TLR4 antibody.

#### Arterial BP measurement

Mean arterial pressure (MAP) was gauged with a sterile catheter inserted into the femoral artery and connected to a pressure transducer (model DT-100; Utah Medical Products). To ensure accuracy of basal levels, MAP was recorded for 1 hour in conscious animals.

### Primary cell culture

VSMCs were obtained from the thoracic aorta of two animals via explant method, as previously described^21^. Cells in passage three were used in all experiments. Results are the average of two independent experiments.

### Western blotting

Aortic tissue was collected, cleaned of surrounding perivascular adipose tissue, homogenized in extraction buffer (T-PER + protease inhibitor cocktail), and stored at − 80$^{\circ }$ C. Measurement of overall protein concentration was performed using a BCA protein assay kit. In total, 20 μg of protein was loaded into SDS-PAGE gel with a 10% concentration followed by transfer to a nitrocellulose membrane. A solution of 5% nonfat-dry milk diluted in Tris-buffered with 1% Tween was used to block nonspecific binding sites during one hour at room temperature. Then, we probed the membranes overnight at a temperature 4 $^{\circ }$C with primary mouse monoclonal antibody for TLR4 (Santa Cruz Biotechnology, sc293072; 1:1,000) and primary rabbit polyclonal antibody for MD2 (Abcam, ab24182; 1:1,000). Immunostaining was detected using horseradish peroxidase-conjugated anti-rabbit or anti-mouse IgG (1:10,000) for 1 hour at room temperature under constant agitation. Immunoblots were revealed by the SuperSignal West Femto Substrate (Thermo Fisher Scientific; 34095). Tissue protein levels were normalized to β-actin (Abcam; ab8227; 1:5,000) expression and quantitated by pixel densitometry using ImageJ (NIH, Bethesda, MD, USA).

### RNA-Seq data analysis

We used the Genotype-Tissue Expression dataset (GTEx, version 7) released in September 2017. Selected RNA-Seq data of aortic tissue from white male donors with type 1 diabetes and hypertension were matched (1:1) with non-diabetic and non-hypertensive donors based on sex, race, and age. We were restricted to this demographic group because of sample size limitations of the GTEx dataset. Donors with type 2 diabetes were not considered. We also used pathologist notes to exclude samples that were reported with any level of atherosclerosis in the aorta from the matching set. The final experimental setup consisted of 8 type 1 diabetic/hypertensive and 8 non-diabetic/non-hypertensive RNA-Seq samples. Data processing and matching procedure were performed using a Python script based on Pandas^22^ version 0.23.4.

### Functional studies

The aorta was isolated from anesthetized animals (isoflurane 5%), cleaned of surrounding fat tissue, and mounted in a 5 ml chamber for assessment of vascular function (Danish Myograph Technology, Aarhus, Denmark). A Krebs solution (37 $^{\circ }$ C) was used to immerse the rings (2 mm) together with a combination of 95% $\hbox {O}_{2}$/ 5% $\hbox {CO}_{2}$. For preload, the rings were applied a stretch of 15 mN/mm, and then, left for 60 min to return to equilibrium. A KCl solution (120 mmol/l) was used to assess the viability of the preparation. Rings were then carefully rinsed to baseline tension, followed by an additional 30 min equilibrium period. Viable rings from CTL and STZ animals were then incubated with CLI095 (InvivoGen, $10^{-5}$ mol/l; DMSO diluted) or L48H37 (Sigma Aldrich, $10^{-5}$ mol/l, DMSO diluted) for 30 minutes to form the following experimental groups: (1) CTL, (2) CTL + CLI095, (3) CTL + L48H37, (4) STZ, (5) STZ + CLI095, and (6) STZ + L48H37. Then, we applied phenylephrine in doses ranging from $10^{-9}$ to $10^{-4}$ mol/l and measured the concentration-response.

In another set of experiments, before conducting the functional studies, we mechanically removed the endothelium from aortic rings. To confirm the absence of the endothelium, after testing for the viability of the preparation, we contracted the rings with phenylephrine ($10^{-5}$ mol/l) and we stimulated them with acetylcholine ($10^{-5}$ mol/l). The success of our approach was determined by the absence of acetylcholine-induced relaxation after 5 minutes of stimulation. Rings were then rinsed to baseline tension and processed as described above.

### ROS detection

#### In vitro ROS detection

Plates of 96 wells were seeded with VSMCs (2560 cells/well, 150 μl). Upon reaching a confluence of 80%, cells were serum deprived for 12 h. Then, they were incubated with CLI095 (InvivoGen, $10^{-5}$ mol/l; DMSO diluted) or L48H37 (Sigma Aldrich, $10^{-5}$ mol/l, DMSO diluted) or vehicle for 24 h with or without high glucose (33 mmol/l). Mannitol (33 mmol/l) was used as an osmotic control. AngII ($10^{-7}$ mol/l) was used as a positive CTL as it is a recognized endogenous agonist of the TLR4–MD2 complex^16,17^. ROS generation was indirectly measured using the dihydroethidium (DHE; ThermoFisher Scientific, D11347) probe. Briefly, DHE staining was diluted in DMEM medium (10 μmol/l) instantaneously before use, and 100 μl was applied to each well. Then, cells were incubated for 30 min (37 $^{\circ }$C) in the absence of light. Wells were rinsed with 1 $\times$ PBS and visualized in a confocal microscope under a 20 $\times$ objective lens. Two images were acquired in each well. The computer program ImageJ (NIH, Bethesda, MD, USA) was used to load and analyse the images. Before measuring the fluorescence intensity, we converted each image to an 8-bit format and we removed the background.

#### In situ ROS detection

The aorta was cleansed of surrounding fat tissue immersed in cold Krebs solution. Then, rings were incubated for 6 h with CLI095 (InvivoGen, $10^{-5}$ mol/l; DMSO diluted) or L48H37 (Sigma Aldrich, $10^{-5}$ mol/l, DMSO diluted) in an isolated muscle bath myograph chamber filled with Krebs’s solution (37 $^{\circ }$C) and supplied with a mixture of 95% $\hbox {O}_{2}$ / 5% $\hbox {CO}_{2}$. CTL rings were incubated with only vehicle. Then, transverse sections of aorta (10 μm) were obtained in a cryostat. Slides were then incubated in PBS for 10 min to rinse them from freezing medium. DHE probe was diluted in Krebs solution (10 μmol/l; ThermoFisher Scientific, D11347) instantaneously before use, and 100 μl was applied to each section. Slides were then incubated for 30 min at 37 $^{\circ }$C in the dark. Slide images were obtained and processed using the aforementioned procedure.

### Statistical analysis

Data points are shown as means ± SEM. For MAP and confocal analysis, statistical significance was calculated using one-way ANOVA. Student’s t-test was used to compute significance of differences in the Western blotting and in the focused RNA-Seq data analysis. Pearson correlation ($\rho$) was computed when appropriated. For functional studies, contraction was computed relative to the maximal tone elicited by KCl, which was taken as 100% in each sample. The least squares method was used to fit the concentration-response curves. The test applied was the two-way ANOVA with Bonferroni correction. Statistical significance was considered for values of p < 0.05.

## Results

STZ-induced diabetic rats displayed augmented levels of glucose and reduced body weight compared with control animals (Table 1). As we previously reported, the chronic neutralising treatment of the TLR4–MD2 complex did not change these outcomes in control nor in diabetic animals^19^.

**Table 1:** Animal profile. Values are shown as mean ± SEM, n = 4. *p < 0.05 vs. control.

Group	Body weight (g)		Glucose levels (mg/dl)	
	14 days	28 days	14 days	28 days
CTL	318.25 ± 9.36	399.75 ± 6.75	83.5 ± 3.61	78.25 ± 4.55
CTL + anti-TLR4	338 ± 10.23	392.25 ± 8.66	81 ± 3.24	81.25 ± 3.49
STZ	242.25 ± 6.35*	279.5 ± 5.42*	370 ± 10.88*	387.75 ± 10.88*
STZ + anti-TLR4	258.25 ± 5*	286 ± 8.68*	384.75 ± 9.89*	398 ± 11.96*

### Chronic blockade of the TLR4–MD2 complex reduces MAP in STZ-induced diabetic rats: implications for type 1 diabetes-induced hypertension

**Figure 1:**
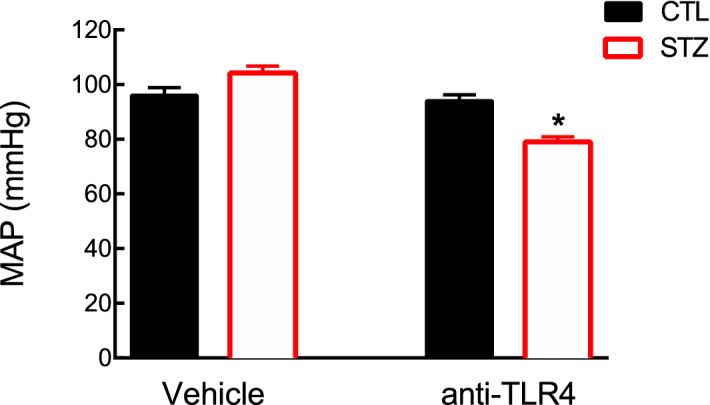
Systemic blockade of the TLR4-MD2 complex with a neutralizing antibody lowers mean arterial pressure in STZ-induced diabetic animals. The mean arterial pressure (mmHg) was determined in control and STZ-induced diabetic (65 mg/kg, i.p., 28 days) rats subjected to a TLR4 antibody (1 μg/daily, i.p.) in the last 14 days of the treatment. Values are shown as mean ± SEM, n = 4. *p < 0.05 vs. control and STZ treated with vehicle.

Considering the alleged role played by TLR4 in BP modulation in animal models of hypertension, we sought to investigate whether systemic blockade of this receptor with a neutralising TLR4 antibody would impact MAP in STZ-induced diabetic animals (Fig. 1). Previous works have demonstrated that STZ-induced diabetic rats may have a predisposition to developing a mild elevation in BP^23–25^. In this study, we observed that diabetic animals only presented a tendency towards higher MAP values compared with control animals (mmHg: 104.3 ± 2.46 vs. 96 ± 2.82, p > 0.05), which corroborates the idea that over time these animals might become hypertensive. More importantly, we found that chronic blockade of TLR4 lowers MAP in diabetic animals (mmHg: 79 ± 1.87 vs. 104.3 ± 2.46; treated vs. non-treated), suggesting that this receptor might contribute to prolonged type 1 diabetes-induced hypertension.

### Differential modulation of the TLR4–MD2 complex in the aorta: insights from diabetic rats and human type 1 diabetic/hypertensive donors

**Figure 2:**
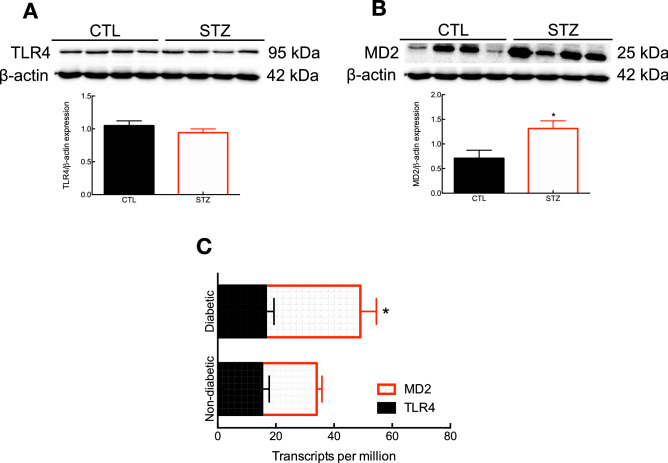
Type 1 diabetes modulates the expression levels of MD2, but not TLR4 in the aorta. Immunoblots and densitometry of TLR4 (**A**) and MD2 (**B**) in control and STZ-induced diabetic rats (28 days). Expression levels for TLR4 and MD2 were normalized to β-actin (n = 4). Uncropped membranes are shown in Supplementary Figure S1. (**C**) Normalized mRNA expression levels in TPM for TLR4 and MD2 (n = 8). Data points are shown as mean ± SEM. *p < 0.05 vs. control group.

We assessed the expression levels of TLR4 and its co-adaptor MD2 in the aorta of control and STZ-induced diabetic rats by Western blotting. Surprisingly, we observed that, compared to control animals, diabetic rats present similar levels of TLR4 after 4 weeks (Fig. 2A). On the other hand, these animals display a significant increase in the expression levels of MD2 (1.85-fold, Fig. 2B), which could be a mechanism mediating TLR4 activation, and consequently, vascular dysfunction in these animals.

Next, we used focused RNA-Seq data analysis to investigate the interplay between type 1 diabetes/hypertension and the TLR4–MD2 complex in human aortic tissues. We observed that, compared with matched non-diabetic/non-hypertensive donors (age: 58.12 ± 2.6 years; and, BMI: 25.84 ± 0.99), the presence of type 1 diabetes/hypertension (age: 58.12 ± 2.6 years; and, BMI: 26.97 ± 0.90) associates with an increase in the expression levels of MD2 but not TLR4 (Fig. 2C).

### Blockade of the TLR4–MD2 complex attenuates vascular hypercontractility (%KCl) in the aorta of STZ-induced diabetic rats

**Figure 3:**
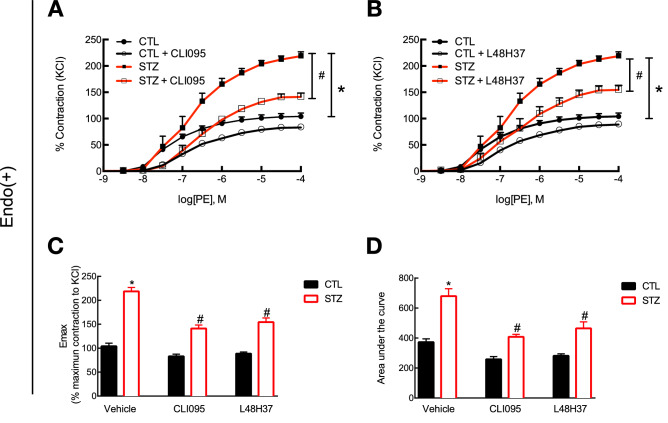
TLR4–MD2 complex mediates vascular hypercontractility in the aorta of STZ-induced diabetic rats. Intact aortic rings from control and STZ-induced diabetic animals were ex vivo incubated with an inhibitor for (**A**) TLR4 (CLI095, $10^{-5}$ mol/l, DMSO diluted) or (**B**) MD2 (L48H37, $10^{-5}$ mol/l, DMSO diluted) for 30 min in an isolated chamber filled with Krebs’s solution (37 $^{\circ }$C) and supplied with a mixture of 95% $\hbox {O}_{2}$/5% $\hbox {CO}_{2}$. Functional studies were performed under isometric force recording in a wire myograph. (**C**) $\hbox {E}_{\mathrm{max}}$ and (**D**) total area under the curve. Values are shown as mean ± SEM, n = 6–8. *p < 0.05 vs. control and ^#^p < 0.05 vs. STZ vehicle.

We performed functional studies in the aorta of diabetic animals with and without pharmacological inhibitors for TLR4 or MD2 (CLI095 or L48H37, $10^{-5}$ mol/l). We observed that diabetic animals presented an increased contractile response to phenylephrine, an $\alpha$-1 adrenergic agonist, compared to control animals ($\hbox {E}_{\mathrm{max}}$ (%KCl): 203.50 ± 6.96 vs. 100.90 ± 6.02; pEC50: 6.77 ± 0.11 vs. 7.30 ± 0.09; and, AUC: 679.78 ± 49.26 vs. 372.42 ± 22.59) and that acute blockade of TLR4 attenuated the shift to the left in the concentration-response curve to phenylephrine as well as the maximum contraction response elicited by the drug ($\hbox {E}_{\mathrm{max}}$ (%KCl): 131.52 ± 5.21 vs. 203.50 ± 6.96; pEC50: 6.49 ± 0.07 vs. 6.77 ± 0.11; and, AUC: 408.66 ± 16.64 vs. 679.78 ± 49.26; inhibitor vs. vehicle, Fig. 3A). Similar results were observed in rings incubated with a small molecule inhibitor for MD2 ($\hbox {E}_{\mathrm{max}}$ (%KCl): 144.21 ± 9.84 vs. 203.50 ± 6.96; pEC50: 6.60 ± 0.14 vs. 6.77 ± 0.11; and, AUC: 465.28 ± 42.87 vs. 679.78 ± 49.26; inhibitor vs. vehicle, Fig. 3B), which confirms that the TLR4–MD2 complex participates in the mechanisms associated with hypercontractility in isolated diabetic vessels.

**Figure 4:**
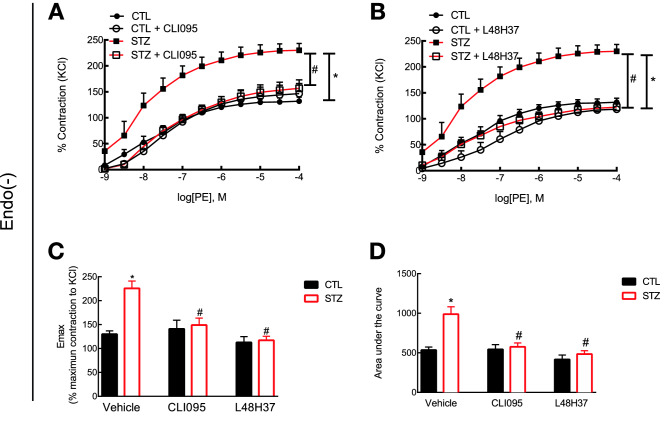
TLR4–MD2 complex mediates vascular hypercontractility in the aorta of diabetic rats independently of the endothelial layer. Denuded aortic rings from control and STZ-induced diabetic animals were ex vivo incubated with an inhibitor for (**A**) TLR4 (CLI095, $10^{-5}$ mol/l, DMSO diluted) or (**B**) MD2 (L48H37, $10^{-5}$ mol/l, DMSO diluted) for 30 min in an isolated chamber filled with Krebs’s solution (37 $^{\circ }$C) and supplied with a mixture of 95% $\hbox {O}_{2}$/5% $\hbox {CO}_{2}$. Functional studies were performed under isometric force recording in a wire myograph. (**C**) $\hbox {E}_{\mathrm{max}}$ and (**D**) total area under the curve. Values are shown as mean ± SEM, n = 4–5. *p < 0.05 vs. control and ^#^p < 0.05 vs. STZ vehicle.

Endothelial cells release endothelium-derived relaxation and/or contractile factors^26^ and express the TLR4–MD2 complex^27^. Therefore, we next performed functional studies in the absence of the endothelial layer. As expected, denuded rings from diabetic animals presented a shift to the left in their concentration-response curve to phenylephrine with no difference in the maximum response elicited by the drug [$\hbox {E}_{\mathrm{max}}$ (%KCl): 225.79 ± 15.14 vs. 203.50 ± 6.96; pEC50: 8.47 ± 1.04 vs. 6.77 ± 0.11; and, AUC: 987.88 ± 94.06 vs. 679.78 ± 49.26; endo(−) vs. endo($+$)]. More importantly, blockade of TLR4 in diabetic denuded rings prevented not only the shift to the left in the concentration-response curve but also the hypercontractility of the preparation ($\hbox {E}_{\mathrm{max}}$ (%KCl): 149.02 ± 14.48 vs. 221.63 ± 13.71; pEC50: 7.74 ± 0.49 vs. 8.47 ± 1.04; and, AUC: 575.76 ± 40.05 vs. 943.63 ± 88.63; inhibitor endo(−) vs. vehicle endo(−), Fig. 4A). Comparable results were observed in denuded rings incubated with the pharmacological inhibitor for MD2 ($\hbox {E}_{\mathrm{max}}$ (%KCl): 117.13 ± 13.83 vs. 221.63 ± 13.71; pEC50: 7.05 ± 0.35 vs. 8.47 ± 1.04; and, AUC: 484.66 ± 42.35 vs. 943.63 ± 88.63; inhibitor endo(−) vs. vehicle endo(−), Fig. 4B). Such data suggest that the effects of the TLR4–MD2 complex in type 1 diabetes-associated vascular hypercontractility occur independently of the endothelial layer.

### The TLR4-MD2 complex correlates with p22phox in the aorta of human type 1 diabetic/hypertensive donors

**Figure 5:**
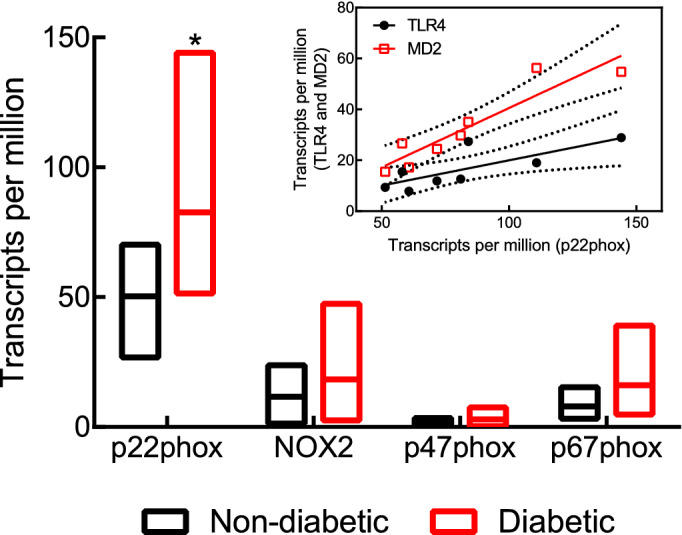
The TLR4/MD2 complex strongly associates with p22phox in the aorta of human type 1 diabetic/hypertensive donors. Normalized mRNA expression levels in TPM for p22phox, NOX2, p47phox, and p67phox. Inset graphic showing the Pearson correlation $\rho$ between p22phox vs. TLR4 and as p22phox vs. MD2. n = 8, *p < 0.05 vs. control group.

NADPH oxidases are important originators of ROS in vascular tissues^28^. Therefore, we investigated the effect of diabetes/hypertension in the expression levels of these enzymes using a human transcriptomic dataset of aortic tissue. We observed that human type 1 diabetic/hypertensive donors have higher levels of the subunit p22phox compared to non-diabetic/non-hypertensive donors (Fig. 5, p < 0.05). These donors also present a tendency towards higher levels for the subunits NOX2, p47phox, and p67phox (Fig. 5, p > 0.05). A similar pattern was also observed for NOX1, but with a very low magnitude of expression (TPM: 0.15 ± 0.03 vs. 0.21 ± 0.04; diabetic/hypertensive vs. non-diabetic/non-hypertensive, p > 0.05). Regarding NOX4, diabetic/hypertensive samples have a significant reduction in its expression levels (TPM: 29.92 ± 5.26 vs. 49.32 ± 6.35; diabetic/hypertensive vs. non-diabetic/non-hypertensive, p < 0.05). Interestingly, we found a strong positive correlation between p22phox and TLR4 ($\rho$= 0.77, p < 0.05) as well as p22phox and MD2 ($\rho$= 0.92, p < 0.05).

### Blockade of the TLR4-MD2 complex attenuates oxidative stress in VSMCs subjected to high glucose and in the aorta of STZ-induced diabetic rats

**Figure 6:**
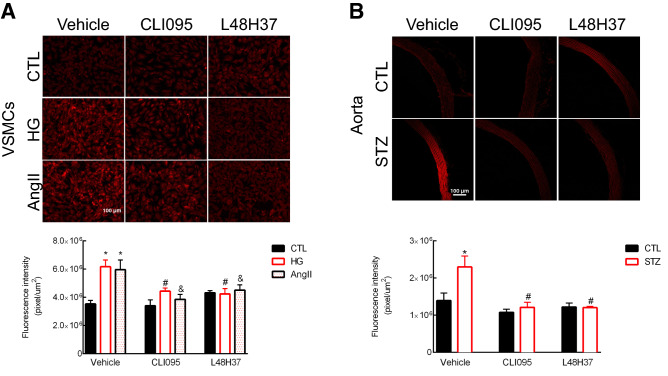
TLR4–MD2 complex induces ROS generation in VSMCs challenged with high glucose and in the aorta of diabetic rats. ROS levels were indirectly measured with the fluorescent probe DHE (10 μmol/l) in VSMCs subjected to high glucose or AngII (positive CTL) (**A**) and in the aorta of STZ-induced diabetic rats (**B**). Representative images are shown with background removed. VSMCs were incubated with CLI095 or L48H37 for 24 h with or without high glucose (33 mmol/l) or AngII ($10^{-7}$ mol/l). Cells were then incubated with the DHE probe for 30 min at 37 $^{\circ }$C in the dark. Data are expressed as mean ± SEM, n = 6. Aortic rings from control and diabetic animals were ex vivo incubated with an inhibitor for TLR4 (CLI095, $10^{-5}$ mol/l, DMSO diluted) or MD2 (L48H37, $10^{-5}$ mol/l, DMSO diluted) for 6 h in an isolated chamber filled with Krebs’s solution (37 $^{\circ }$C) and supplied with a mixture of 95% $\hbox {O}_{2}$/5% $\hbox {CO}_{2}$. Data points are shown as mean ± SEM, n = 4–5. *p < 0.05 vs. control, ^#^p < 0.05 vs. HG or STZ vehicle, and ^&^p < 0.05 vs. AngII vehicle.

ROS affects the mechanisms of vascular function and dysfunction in healthy and diseased states. Therefore, we next investigated whether blockade of TLR4 or MD2 would impact superoxide generation, the main ROS subprodcut of NADPH oxidases, in VSMCs challenged with high glucose or AngII (positive CTL) and in the aorta of diabetic rats. We report that VSMCs subjected to high glucose or AngII present increased levels of ROS compared to control samples and that acute blockade of TLR4 or MD2 attenuates oxidative stress in these cells (Fig. 6A). Blockade of TLR4 or MD2 elicited similar results in our ex vivo set of experiments in the aorta of diabetic rats (Fig. 6B), which highlights the TLR4–MD2 complex as an essential modulator of vascular oxidative stress during type 1 diabetes.

## Discussion

In this study, we showed for the first time, that systemic blockade of the TLR4–MD2 complex with an anti-TLR4 neutralizing antibody significantly lowers BP in the STZ-induced diabetic model (Fig. 1), which is an animal model of type 1 diabetes that may have a predisposition to developing a mild elevation in BP^23–25^. High BP is a frequent comorbidity of type 1 diabetes^2^. Therefore, the ADA endorses values of less than 130/80 mmHg for younger type 1 diabetic patients^4^, primarily because of the increased risk of diabetic kidney disease.

There is mounting evidence showing an interplay between TLR4 and AngII, a key peptide of the RAS, in the major organs that control BP (for a review, see^29^). However, only recently, it was demonstrated that hyperglycemia induces RAS activation via modulation of the MD2–TLR4–MAPK axis in the diabetic kidney. Blockade of MD2 suppresses the expression levels of the angiotensin-converting enzyme (ACE), angiotensin type 1 receptors, and AngII in cultured kidney cells^15^, which could be a mechanism influencing BP outcomes in these animals, mainly because the RAS system is a critical regulator of this hemodynamic parameter in healthy and diseased states. Interestingly, systemic blockade of TLR4 in AngII-infused animals does not lower BP^30^. Similar outcomes in this animal model were also observed in animals with a deficiency in $\hbox {TLR4}_{\mathrm{lps-d}}$^31,32^. In this context, we argue that (a) if the mechanism by which systemic TLR4 blockade lowers BP in diabetic animals involves suppression of the RAS system, in animals receiving exogenous AngII, the treatment would not impact BP; (b) blockade of TLR4 reduces vascular contractility in small resistance arteries^10^, which directly contributes to BP regulation by modifying total peripheral resistance^33^; and, (c) although diabetes and hypertension have many overlapping mechanisms, they are independent diseases.

While previous studies have demonstrated that hyperglycemia modulates the expression levels of TLR4 in the aorta of STZ-induced diabetic animals^34,35^, we did not observe differences in the expression levels of this receptor (Fig. 2A). Studies that reported increased levels of TLR4 in the aorta were conducted at later stages of the disease (8 and 16 weeks) and we harvested samples after 4 weeks. Considering that diabetes is a progressive disease, especially in non-treated animals, it is reasonable to speculate a time dependency in the expression levels of TLR4 in this tissue. Here, we also showed for the first time that type 1 diabetes affects MD2 levels in the aorta of diabetic animals (Fig. 2B). MD2 is a co-adaptor for TLR4 in innate immunity^14^ acting as a gateway between the receptor and its primary exogenous ligand, lipopolysaccharide^36^ and emerging evidence suggests that MD2 also plays a role in recognition of endogenous molecules, such as AngII^16,17^, HMGB1^37^, and HSP70^38^, which highlights this co-adaptor as a strategic target within this molecular pathway. Interestingly, we also observed an increase in MD2 levels in the aorta of type 1 diabetic/hypertensive donors, but not TLR4 (Fig. 2C). Post-translational modifications regulate TLR4 traffic to the plasma membrane^39^, which influences the receptor activity. Indeed, a previous report has shown that N-linked glycosylation of TLR4 and MD2 are critical for maintaining the functionality of this complex^40^. In light of this information and based on our results, we argue that a non-increase in TLR4 expression does not translate into a non-increase in receptor activity. It is also important to consider that we did not have access to the treatment regimen of donors, which might affect the observed outcomes.

Type 1 diabetes-induced hypertension increases the risk of CVDs in diabetic patients^4^. Although the underlying molecular mechanisms of this process are not entirely understood, it has been suggested that TLR4 activation contributes to the pathophysiology of diabetic vascular complications^21^, including dysfunctionality of small resistance arteries^10^. Here, we expand the contributions of TLR4 to the mechanisms of diabetic vasculopathies as we show that acute blockade of this receptor attenuates vascular contractility in large conducting vessels isolated from diabetic animals (Fig. 3A). We observed similar results in the presence of an inhibitor for MD2 (Fig. 3B). This is particularly important in this context because it has been recently suggested that vascular dysfunction in large arteries impairs the vessels’ compliance, which might increase the conduction of pulsatility to small resistance arteries^41^. Corroborating our findings, it has been previously reported that mice lacking the MD2 gene are protected against obesity-induced vascular remodeling of the aorta^18^. Because MD2 is an upstream protein of TLR4, it is reasonable to consider that blockade of this co-adaptor should lead to similar outcomes compared to inhibition of TLR4. In line with this assumption, we did not observe difference in the $\hbox {E}_{\mathrm{max}}$ or AUC for phenylephrine in the presence of CLI095 and L48H37 between the control and diabetic groups (Fig. 3C,D, respectively), which suggests that while these inhibitors have independent mechanisms of action, they elicit similar effects in isolated aortic rings, at least, under the conditions investigated in this study.

The endothelium is a dynamic layer that lines the inner side of blood vessels. Its role in modulating vascular tone is well recognized as endothelial cells not only have receptors to a variety of vasoactive substances but also because they release endothelium-derived relaxation and/or contractile factors^26^. Additionally, these cells express the TLR4–MD2 complex^27^, which might crosstalk with the contractile machinery. Therefore, aiming to rule out the endothelial influence, we conducted functional studies in denuded vessels. Our results show that the effects of the TLR4–MD2 complex in vascular contractility are independent of the endothelial layer (Fig. 4A,B). Again, we did not observe differences between the treated groups of CLI095 and L48H37 in control and diabetic animals (Fig. 4C,D).

Pharmacological inhibition of the TLR4–MD2 complex in diabetic vessels might affect many downstream signaling mechanisms involved in vascular function and dysfunction, including ROS. In fact, we observed that inhibition of TLR4 or MD2 significantly attenuates ROS (superoxide) production in VSMCs challenged with high glucose and in the aorta of diabetic rats (Fig. 6A,B). While there is still extensive debate about the mechanism leading to TLR4 activation during type 1 diabetes, it seems that sustained hyperglycemia drives the generation of DAMPs, which, in turn, signal through this receptor leading to an increase in oxidative stress. Specifically, TLR4 might modulate ROS levels by affecting the NADPH oxidase enzyme^31,42,43^. This is particularly important because we also observed that type 1 diabetic/hypertensive human donors have increased levels of p22phox (Fig. 5), a membrane-bound subunit that participates not only in the activation of NADPH oxidase 2, but also affects NADPH oxidase 1 and 4. Noteworthy, TLR4 deficiency reduces AngII-induced ROS in kidney homogenates by suppressing p22phox^44^. Still, further research is needed to confirm that, during type 1 diabetes, the TLR4–MD2 complex induces superoxide generation by direct modulation of the p22phox subunit. While NOX1 and NOX2 are primary sources of superoxide in the vasculature^28,45^, NOX4-induced generation of ROS might protect vascular structures^46^, but unfortunately, we observed that human type 1 diabetic/hypertensive donors have a significant reduction in the expression levels of this protein compared with matched controls.

ROS are vasoactive substances that alter smooth muscle tone and contribute to an imbalance in the antioxidant system^47^. Recent evidence suggests that the deletion of MD2 induces the activation of the Nuclear factor erythroid 2-related factor 2 (Nrf2) in the aorta of high-fat diet fed mice^18^, a transcriptional factor that regulates the expression of many antioxidant genes^48^. In this work, authors also demonstrated that in VSMCs exposed to palmitic acid, the inhibition of MD2 with a neutralizing antibody reduces ROS generation by activating AMPK/Nrf2 signaling^18^. Therefore, another important aspect in this interplay that one could further explore is the link between hyperglycemia-induced ROS, the antioxidant system, and the TLR4–MD2 complex, which might provide insights into the mechanisms associated with the disease progression.

Taken together, our findings are a step towards the development of novel strategies in the treatment of diabetes-induced hypertension. Here, we showed that (a) blockade of TLR4 lowers BP in diabetic animals; that (b) type 1 diabetes differentially modulates the TLR4–MD2 complex in the aorta; and, that (c) the TLR4–MD2 complex contributes to vascular dysfunction in the aorta of STZ-induced diabetic animals. Still, further studies are needed to investigate whether this complex would be a suited target in the clinical setting.

## Supplementary information

Supplementary Information.

## Data Availability

All animal data generated or analysed during this study are included in this published article. The RNAseq data that support the findings of this study are available from the database of Genotypes and Phenotypes (dbGaP) but restrictions apply to the availability of these data, which were used under license for the current study (Project No. 78763-1), and so are not publicly available. RNAseq data are however available from the authors upon reasonable request and with permission of dbGaP.
