# Supplementary material for: Blockade of the TLR4–MD2 complex lowers blood pressure and improves vascular function in a murine model of type 1 diabetes
Source: Sci Rep. 2020 Jul 21;10:12032. doi: 10.1038/s41598-020-68919-x (PMC7374604; doi:10.1038/s41598-020-68919-x)
Supplement: Supplementary file 1 — Supplementary Information. [file 41598_2020_68919_MOESM1_ESM.pdf]

Blockade of the TLR4-MD2 complex lowers blood pressure and improves vascular function in a murine model of type 1 diabetes

Amanda Almeida de Oliveira[1], Josemar Faustino[2], R. Clinton Webb[3], and Kenia Pedrosa Nunes[1,*]

[1]Department of Biomedical and Chemical Engineering and Sciences, Florida Institute of Technology, Melbourne, United States; [2]Department of Computer Engineering and Sciences, Florida Institute of Technology, Melbourne, United States; [3]Department of Cell Biology and Anatomy, University of South Carolina, Columbia, United States; [*]knunes@fit.edu

Supplementary information: full length blot of Figure 2 panels A and B.

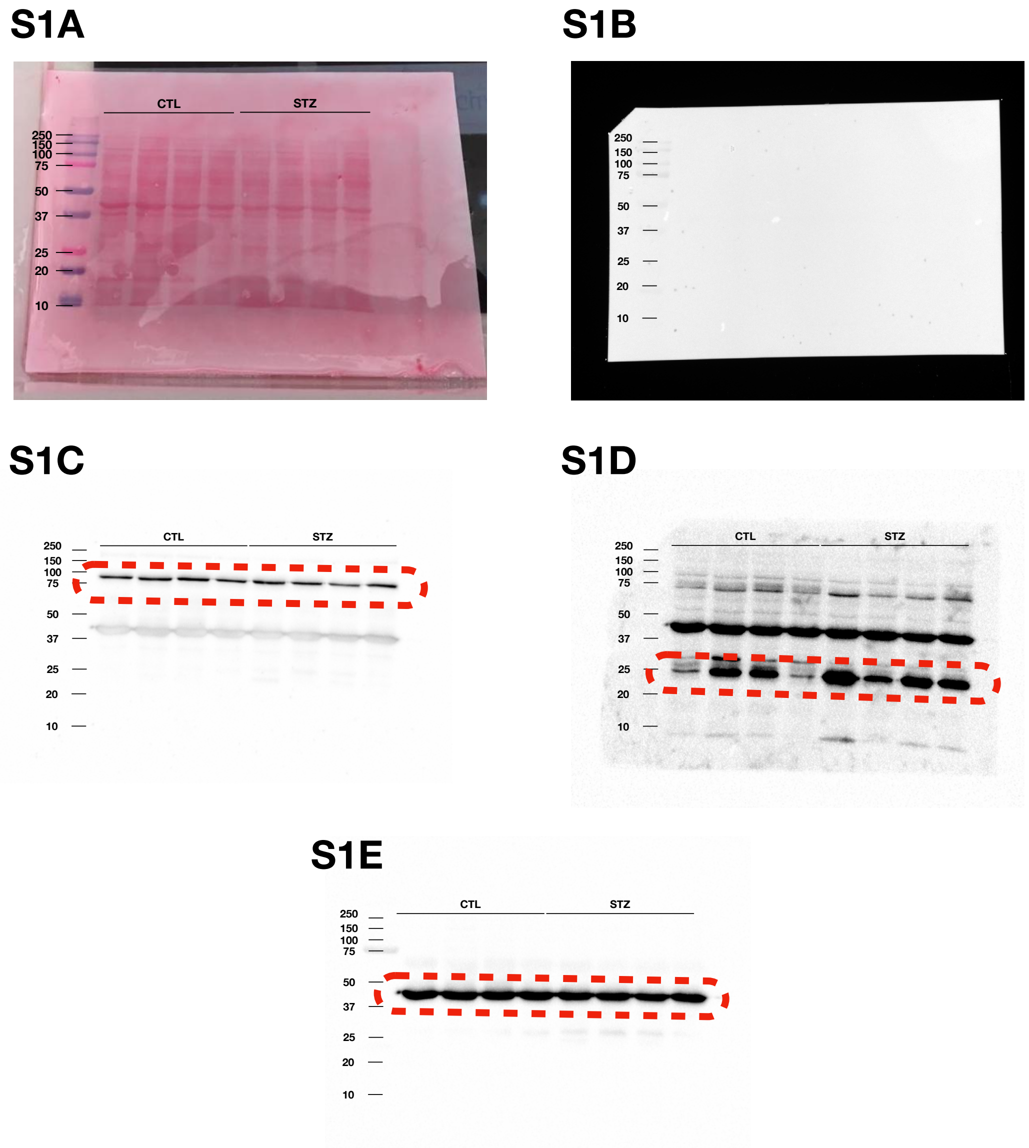

Figure S1. Supplementary uncropped images used in Figure 2. Ponceau staining of the original membrane (A). The same membrane was visualized by epi-luminescence (B) and by chemiluminescence to reveal the bands for TLR4 (95 kDa - C), MD2 (25 kDa - D), and β-actin (42 kDa - E). The red dashed rectangle represents the cropped region used in panels A and B of Figure 2. n=4.
